# Preventive and Prophylactic Mechanisms of Action of Pomegranate Bioactive Constituents

**DOI:** 10.1155/2013/789764

**Published:** 2013-04-30

**Authors:** Monica Viladomiu, Raquel Hontecillas, Pinyi Lu, Josep Bassaganya-Riera

**Affiliations:** ^1^Nutritional Immunology and Molecular Medicine Laboratory, Virginia Bioinformatics Institute, Virginia Tech, Blacksburg, VA 24060, USA; ^2^Center for Modeling Immunity to Enteric Pathogens, Virginia Bioinformatics Institute, Virginia Tech, Blacksburg, VA 24060, USA; ^3^Department of Biomedical Sciences and Pathobiology, Virginia-Maryland Regional College of Veterinary Medicine, Virginia Tech, Blacksburg, VA 24061, USA

## Abstract

Pomegranate fruit presents strong anti-inflammatory, antioxidant, antiobesity, and antitumoral properties, thus leading to an increased popularity as a functional food and nutraceutical source since ancient times. It can be divided into three parts: seeds, peel, and juice, all of which seem to have medicinal benefits. Several studies investigate its bioactive components as a means to associate them with a specific beneficial effect and develop future products and therapeutic applications. Many beneficial effects are related to the presence of ellagic acid, ellagitannins (including punicalagins), punicic acid and other fatty acids, flavonoids, anthocyanidins, anthocyanins, estrogenic flavonols, and flavones, which seem to be its most therapeutically beneficial components. However, the synergistic action of the pomegranate constituents appears to be superior when compared to individual constituents. Promising results have been obtained for the treatment of certain diseases including obesity, insulin resistance, intestinal inflammation, and cancer. Although moderate consumption of pomegranate does not result in adverse effects, future studies are needed to assess safety and potential interactions with drugs that may alter the bioavailability of bioactive constituents of pomegranate as well as drugs. The aim of this review is to summarize the health effects and mechanisms of action of pomegranate extracts in chronic inflammatory diseases.

## 1. Introduction

Pomegranate (*Punica granatum*), an ancient fruit-bearing deciduous shrub, is the predominant member of two species comprising the Punicaceae family [1]. It is a native of the Himalayas in northern India, but it has been cultivated and naturalized throughout the Middle East, the entire European Mediterranean region, the drier parts of southeast Asia, northern and tropical Africa, and to some extent the United States, specifically California and Arizona [2]. Pomegranate's fruit is a large berry characterized by the presence of thin membranes in its interior, which allow the suspension of numerous arils, each surrounded by juice-containing sacs. The fruit can be divided into three parts: the seeds and the juice, which represent about 3 and 30% of the fruit weight, respectively, and the peels, which include the mentioned inner network of membranes [1] with distinct chemical compositions and potential medical benefits.

Pomegranate extracts have been used since ancient times to treat several conditions including parasitic and microbial infections, diarrhea, ulcers, aphthae, hemorrhage, and respiratory complications [3, 4]. Modern applications include hormone replacement therapy and oral hygiene as well as the treatment immune suppression and cardiovascular complications [5]. Moreover, other therapeutic properties such as antitumor, anti-inflammatory, antiviral, antibacterial, antidiarrheal, and antiobesity are currently under investigation. Although pomegranate has been consumed and used as a medicinal food in the Middle East for thousands of years, it has recently gained popularity in the United States [6]. The high antioxidant activity of pomegranate when compared to other fruits and antioxidant beverages, along with its anti-inflammatory and antiobesity properties, has increased the interest in investigating its potential nutraceutical and functional food applications. The number of scientific publications on pomegranate and its health-promoting benefits has increased significantly in the last decade. A PubMed search on November 23, 2012, retrieves 677 publications related to pomegranate, 619 of which have been published within the last ten years and 425 in the last five. Moreover, several pomegranate-containing products, including 100% juices, pomegranate-containing beverages, liquid and powdered polyphenolic extracts of pomegranate, and skin care products containing pomegranate extracts or seed oils, have been recently introduced in the United States market and are being advertised for their beneficial health effects [6]. Therefore, studying the bioactive components of pomegranate and linking them with specific mechanisms of action and health effects holds promise for future therapeutic development of pomegranate-derived natural products. The aim of this review is to summarize the health effects and mechanisms of action of pomegranate extracts in chronic inflammatory diseases.

## 2. Chemical Composition of Pomegranate

Over the last decade, there has been significant progress in investigating the mechanisms of action of pomegranate components in health and disease. Extracts of all the different parts of the fruit seem to have medicinal benefits [1]. Interestingly, recent studies report that even the bark, roots, and leaves of the pomegranate plant also have therapeutic properties [7]. The goal of most of the recent pomegranate studies is to identify its therapeutic constituents. Ellagic acid, ellagitannins (including punicalagins), punicic acid, flavonoids, anthocyanidins, anthocyanins, estrogenic flavonols, and flavones appear to be the most therapeutically beneficial pomegranate components [8], specially ellagitannins, which release ellagic acid when hydrolyzed. Tables 1, 2, 3, and 4 summarize the chemical composition for each of the different parts of the pomegranate fruit.

The pomegranate peels make up about 60% of the fruit, and they are rich in many compounds such as phenolics, flavonoids, ellagitannins (including punicalagins), proanthocyanidin compounds, complex polysaccharides, and many minerals including potassium, nitrogen, calcium, magnesium, phosphorus, and sodium [2]. However, its biological properties are mainly associated with the presence of flavonoids and tannins. The peel is the part of the fruit with the highest antioxidant activity, which is in line with its high content of polyphenols [9, 10]. Moreover, pomegranate peels also show higher antioxidant capacity *in vitro* when compared to other fruits such as mangos, bananas, and coconuts [11]. Ellagitannins present in the pomegranate peel include punicalagin and punicalin, both of which contain the polyphenolic chemical compound gallagic acid, which is the building block for several tannins. Punicalagin can be found in the seeds, peels, and juice of pomegranate, and it is unique to pomegranate. Both punicalagin and punicalin can be hydrolyzed to ellagic acid, a natural phenol with high antioxidant activity, thus prolonging the release of this acid into the blood [8]. Antioxidants are important since they have several important biological properties such as anti-inflammatory and antiaging protection against cholesterol and atherosclerosis [12]. 

Pomegranate juice is a rich source of polyphenols, tannins, anthocyanins, including vitamin C, vitamin E, coenzyme Q10, and lipoic acid [13]. Its main antioxidative compounds are anthocyanins and ellagic acid derivatives, which are the main constituents of the juice, giving the fruit its color [7]. Moreover, anthocyanins have been associated with prevention of cardiovascular disease, obesity, and diabetes [14]. Some differences regarding the phenolic composition are found between natural and commercial juices as well as between juices obtained from the arils alone or from the whole fruit [7]. Nevertheless, pomegranate juice is still the main source for pomegranate ingestion, and its antioxidant levels are greater than in other natural juices [15, 16].

Although pomegranate seeds, which represent about 3% of the fruit weight, have a low polyphenol content and *in vitro* antioxidant capacity, they contain other components that may contribute to pomegranate's health benefits [10, 17]. They are a rich source of lipids, and their oil, which constitutes 12–20% of total seed weight, contains a unique fatty acid profile characterized by high concentration of fatty acids such as linoleic acid (LA) and linolenic acid (LN), as well as other lipids including punicic, oleic, stearic, *α*-eleostearic, *β*-eleostearic, catalpic, gadoleic, arachidic, behenic, and palmitic acids LN (Table 1). Interestingly, punicic acid, which is a conjugated isomer unique to pomegranate oil, constitutes 70–76% of the seed oil. Moreover, seed oil also contains minor amounts of other conjugated trienes such as eleostearic acid and catalpic acid [13]. Other minor components are sterols, steroids, and cerebrosides in the seed oil [8, 18] and proteins, fibers, vitamins, and minerals in the whole seed [13]. 

The relationship between the chemical composition of pomegranate and its respective therapeutic effects is still not fully understood. However, significant progress has been made in the last decade in the establishment and identification of chemicals responsible for certain pharmacological mechanisms. Although ellagic acid, which presents both antioxidant [19] and anticarcinogenic [20] properties, is thought to be the main compound responsible for pomegranate health beneficial effects, recent studies suggest that the synergistic action of several pomegranate constituents is superior to ellagic acid alone in the suppression of prostate cancer [21, 22]. Therefore, and although ellagic acid is at the forefront of pomegranate research, one needs to be cautious and avoid ignoring other compounds present within pomegranate juice, peel, and seeds. Moreover, the health effect of pomegranate can vary due to geographical region, harvesting, and season, which can alter the fruit composition [23]. Regardless of its composition, not all pomegranate-derived compounds elicit beneficial effects when ingested since some of them might not be metabolized and absorbed. Therefore, there is a need to study the metabolism and bioavailability of the pomegranate mixtures.

## 3. Pomegranate Constituent Bioavailability

The high antioxidant capacity of pomegranate has been mostly attributed to its high levels of polyphenolic compounds, especially ellagitannins. However, little is known about the metabolism and bioavailability of ellagitannins from food sources. Therefore, there is a need to provide a link between these compounds and the health effects related to antioxidant activity. Several human trials, as well as animal studies, have studied the bioavailability, absorption, and metabolism of pomegranate. In a case study, 31.9 ng/mL of ellagic acid and its metabolites were detected in the plasma of an individual subject one hour after the ingestion of 180 mL pomegranate juice. Plasma was cleared 4 hours after consumption, suggesting that ellagic acid from food is absorbed in humans [24]. In a follow-up study, the rapid absorption and plasma clearance of ellagitannins was confirmed, as well as the persistent excretion of urolithin metabolites in the urine up until 48 hours after pomegranate juice ingestion [25], which present significant antioxidant and anti-inflammatory properties *in vitro *[26, 27]. Furthermore, no difference in bioavailability was found between pomegranate juice, liquid extract, or powdered extracts as indicated by the levels of ellagic acid and its metabolites in plasma [28]. In contrast, no ellagic acid, punicalagin, anthocyanins, or any of their degradation products was found in plasma 13 days after the administration of 1 L pomegranate juice containing 4.37 g/L punicalagins and 0.49 g/L anthocyanins for 5 days [29]. However, pomegranate juice metabolites including urolithin A, urolithin B, and a third undetermined metabolite were discovered in the plasma. Moreover, these 3 metabolites were present in the urine after 24 hours along with an aglycone metabolite corresponding to each of the plasma metabolites. Based on the timing of the appearance of the metabolites in plasma and urine samples, which occurred 3-4 days after juice ingestion, and along with previous work on punicalagin bioavailability in rats [30, 31], the authors suggested colonic microbial metabolism of pomegranate juice polyphenols. Finally, healthy individuals were placed on a polyphenol- and antioxidant-free diet from 3 days prior the administration of a 800 mg capsuled pomegranate extract containing 330.4 mg punicalagins and 21.6 mg ellagic acid [32]. Results from this third study also demonstrate a significant increase in the levels of ellagic acid in plasma 1 hour after extract administration, as well as an increased antioxidant capacity 0.5, 1, and 2 hours after ingestion.

Using an acorn-fed Iberian pigs model system, 31 ellagitannin metabolites were found, but only urolithin A, urolithin B, dimethyl ellagic acid, and their metabolites were detected in plasma [33]. Urolithin metabolites are also absorbed in mouse models, with high levels accumulated in the prostate, the colon, and other intestinal tissues [34]. In line with these animal studies, urolithin A glucuronide, urolithin B glucuronide, and dimethylellagic acid were detected in human prostate tissues 3 days after the administration of pomegranate juice [35]. Therefore, it seems that ellagitannins are hydrolyzed in the stomach, where some ellagic acid is absorbed into circulation. The rest of ellagic acid is metabolized to urolithin derivatives by colonic microflora, and the less polar urolithin derivatives are then absorbed into circulation and further metabolized to glucuronides [6]. Therefore, pomegranate polyphenolic compounds may exert their effects in different ways: they might be absorbed and enter the bloodstream acting directly as antioxidants, or they can be digested by the gut microflora resulting in other biologically active compounds.

## 4. Mechanisms of Action and Health Effects

**Figure 1:**
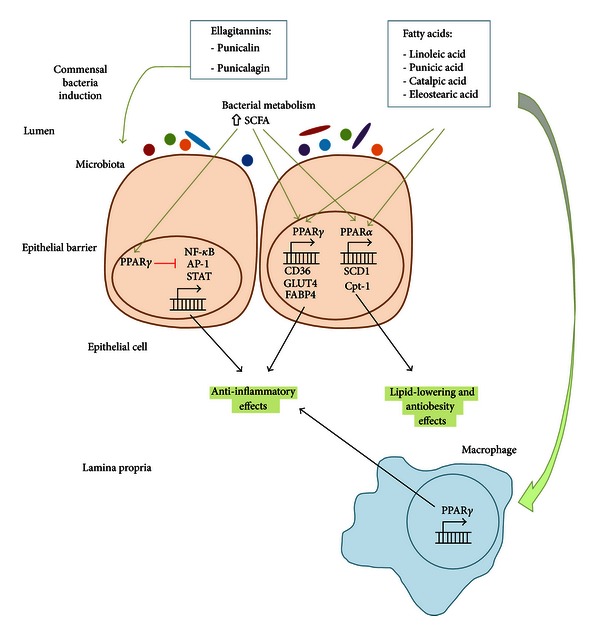
Anti-inflammatory and antiobesity effects of pomegranate constituents. Punicalin and punicalagin are able to increase the bacterial production of short chain fatty acids (SCFAs) by inducing the growth and metabolism of commensal bacteria. SCFAs are then absorbed and activate peroxisome proliferator-activated receptor *γ* (PPAR*γ*), which blocks the transcription of pro-inflammatory molecules by NF-*κ*B, AP-1 and STAT, thus resulting in anti-inflammatory effects. PPAR*γ* can also be activated by fatty acids including linoleic acid, punicic acid, catalpic acid, and stearic acid in both epithelial cells and macrophages. Such fatty acids are also able to activate PPAR*α* resulting in lipid-lowering and antiobesity effects.

The adult human gut contains about 100 trillion microbial organisms [36]. The composition of the human gut microbiota has been associated with both health improvement and the development of several diseases since changes in its composition can modulate the induction of regulatory versus effector immune responses at the mucosal surfaces. Commensal bacteria provide the host with colonization resistance against pathogens, stimulate the host immune system, prevent food allergies and tumors, produce vitamins, metabolize cholesterol and other lipids, and increase mineral bioavailability [37, 38]. However, the overgrowth of these normally beneficial bacteria can cause acute and chronic intestinal diseases and has also been associated with cancer, aging, and obesity [39]. Ellagitannins contained in pomegranate interact with the gut microflora. Pomegranate byproducts and punicalagins inhibit the growth of certain pathogenic *Clostridia* species, *Staphylococcus aureus*, and *Pseudomonas aeruginosa* but increase the growth of *Bifidobacterium breve* and *Bifidobacterium infantis* as well as the production of short chain fatty acids [37, 40], which have been shown to elicit beneficial effects through the activation of peroxisome proliferator-activated receptors (PPARs). PPARs are the receptors for endogenous lipid molecules (i.e., prostaglandins or hydroxy-containing PUFA such as 12/15-hydroxyeicosatetraenoic (HETE), 13-hydroxyoctadecadienoic (HODE)) and molecular targets for drugs against type 2 diabetes [41–43] and represent promising new targets for the treatment and prevention of inflammatory disorders [44, 45]. PPARs are ligand-induced transcription factors that belong to the nuclear hormone receptor superfamily with 48 members identified in the human genome. They regulate gene expression by binding with Retinoid X Receptor (RXR) as a heterodimeric partner to specific DNA sequence elements named Peroxisome Proliferator Response Element (PPRE) [46]. PPARs are the main modulators of lipid and carbohydrate metabolism [47]. Functionally, PPARs regulate inflammation, immunity, and metabolism [48]. There are three known PPAR isoforms: *α*, *β* or *δ*, and *γ*, which differ in their tissue distribution and functional activity [49]. PPAR*α* is important in the clearance of circulating or cellular lipids via the regulation of gene expression involved in lipid metabolism in liver and skeletal muscle [50]. PPAR *β*/*δ* is involved in lipid oxidation and cell proliferation [51], whereas PPAR*γ* promotes adipocyte differentiation to enhance blood glucose uptake [50]. Moreover, ligand-induced activation of PPAR*γ* can antagonize the activity of proinflammatory transcription factors such as nuclear factor kappa-light-chain-enhancer of activated B cells (NF-*κ*B), signal transducer and activator of transcription (STAT), and activator protein (AP)-1 [52] (Figure 1). Both PPAR*γ* and PPAR*δ* serve as targets for the treatment of inflammatory and immune-mediated diseases because of the role they play in maintaining homeostasis and suppressing inflammation [53, 54]. Their activation and expression is controlled by a diverse set of natural and synthetic molecules, including nutrients, nonnutrient endogenous ligands, and drugs (i.e., thiazolidinediones (TZDs) and fibrates) [55]. However, rosiglitazone and other PPAR*γ* agonists of the TZD class of antidiabetic drugs are unlikely to be adopted by gastroenterologists due to associated side effects [56] including hepatotoxicity, weight gain, fluid retention leading to edema, and congestive heart failure [57]. Therefore, the use of natural therapeutics able to activate PPARs is a safer alternative to TZDs. In this regard, the administration of PPARs naturally occurring agonists holds promise for the treatment of a wide range of diseases including obesity, diabetes, and intestinal inflammation [55, 58–60].

### 4.1. Pomegranate Constituents in the Prevention of Obesity and Insulin Resistance

**Figure 2:**
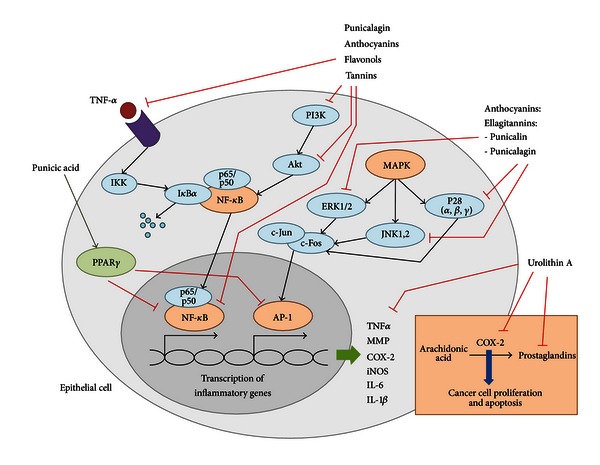
Anticarcinogenic effects of pomegranate constituents. Several pomegranate constituents including anthocyanins, phenols, ellagitannins (punicalin, punicalagin), and other tannins can reduce the expression of cyclooxygenase 2 (COX-2) through an NF-*κ*B and MAPK pathways dependence. Such components can inhibit phosphatidylinositide 3-kinases (PI3K), protein kinase B or Akt, or NF-*κ*B directly and result in decreased transcription of inflammatory genes such as tumor necrosis factor *α* (TNF-*α*), interleukin 6 (IL-6), and IL-1*β* among others. They can also inhibit MAPK-induced phosphorylation of ERK1/2, JNK1,2,3 and p38 which finally result in the inhibition of activation protein 1 (AP-1), another transcription factor regulating the expression of pro-inflammatory molecules. Inhibited COX-2 expression leads to reduced cell proliferation and apoptosis as well as decreased production of prostaglandins, which are important inflammatory mediators.

Naturally occurring compounds that simultaneously activate PPAR*α*, PPAR*β*/*δ*, and PPAR*γ* are promising therapeutic approaches to treat metabolic syndrome, obesity, and diabetes since they reduce serum triglyceride and glucose levels, improve insulin sensitivity, and increase the levels of the high-density lipoprotein (HDL) [61]. Pomegranate seed oil activates PPAR*γ* in mice, thus resulting in an alteration of adiposity, lower leptin levels, and increased adiponectin when fed a high-fat diet (HFD) [62]. PPAR reporter activity data demonstrated a dose-dependent increase in the ability of punicic acid to activate PPAR *α* and *γ* in 3T3-L1 cells [62]. Furthermore, the similar patterns of PPAR *α* and *γ* activation by punicic acid and pomegranate seed oil suggested that the effects of pomegranate seed oil on PPAR *α* and *γ* reporter activities were mediated by punicic acid. Punicic acid robustly bound and activated PPAR *α* and *γ*, thus upregulating PPAR *α* and its responsive genes (Stearoyl-CoA desaturase-1, SCD1; Carnitine palmitoyltransferase 1, Cpt-1; and acyl-coenzyme A dehydrogenase) as well as PPAR *γ*-responsive genes expression (CD36 and Fatty Acid Binding Protein4, FABP4) in intra-abdominal white  adipose tissue while suppressing expression of the inflammatory cytokine tumor necrosis factor *α* (TNF-*α*) and NF-*κ*B activation. Moreover, these changes in gene expression correlated with improved fasting glucose concentrations and glucose-normalizing abilities in mice treated with punicic acid. Overall, dietary punicic acid ameliorated obesity-related inflammation and insulin resistance by activating PPAR *γ*, repressing TNF-*α* expression and NF-*κ*B DNA-binding activity in white adipose tissue and liver. However, the loss of PPAR *γ* in immune cells impaired the ability of punicic acid to modulate glucose homeostasis and obesity-related inflammation, thus suggesting that punicic acid ameliorates metabolic and inflammatory changes associated with obesity through a PPAR *γ*-dependent mechanism [62] (Figure 1). In line with these results, pomegranate seed oil has been found to inhibit TNF-*α*-induced neutrophil hyperactivation and protect from experimental colonic inflammation in rats by targeting the p38MAPKinase/Ser345-p47phox axis [63] (Figure 2).

High-fat-diet- (HFD-) fed mice treated with pomegranate seed oil resulted in decreased body weight and fat mass in CD-1 mice when compared to the control group, although no differences were reported regarding the lean mass between both groups [64]. Moreover, pomegranate seed oil administration improved glucose intolerance in HFD-fed mice, thus reducing type II diabetes risk. In line with these results, catalpic acid decreased the accumulation of abdominal white adipose tissue, improved glucose tolerance, and upregulated adipose PPAR*α* in two different mouse models of obesity [62]. Moreover, catalpic acid also increased HDL cholesterol and decreased triacylglycerol and insulin levels in plasma [65]. These changes were associated with an upregulation of PPAR*α* and its responsive genes in white adipose tissue of mice fed catalpic acid-supplemented diets. In contrast to punicic acid that modulates obesity comorbidities such as insulin resistance, diabetes, or systemic inflammation, catalpic acid has a direct effect in reducing adipose tissue accumulation. The treatment of diabetic mice with PPAR*α* agonists also normalizes circulating fatty acid and triacylglycerol levels and decreases myocardial fatty acid oxidation [66]. Therefore, the use of pomegranate-derived compounds and fatty acids holds promise as prophylactic interventions for obesity and diabetes since some of them are PPAR*α* and PPAR*γ* activators that contribute to correct the abnormalities of lipid metabolism and regulate inflammatory responses. Mounting evidence demonstrates that pomegranate-derived compounds including punicic acid and catalpic acid can act on pharmacologic targets such as PPARs and elicit their therapeutic properties in mice. Specifically, punicic acid can be used to regulate blood sugar levels and control intestinal inflammation, whereas catalpic acid can be used for antiobesity and lipid-lowering applications (Figure 1).

Other pomegranate constituents have been investigated individually or in combination in relation to their lipid-normalizing effects. HFD-fed obese mice treated with dietary gallic acid, linolenic acid, or their mixture showed a body weight loss of 12.8%, 6.8%, and 12.20% after 7 weeks, respectively [67]. Moreover, obese mice showed decreased total cholesterol, triacylglycerols, HDL cholesterol, and low-density lipoprotein (LDL) cholesterol when compared to control mice regardless of the treatment. Therefore, these results suggest that gallic acid and linolenic acid present lipid-lowering effects and may protect patients from obesity and other hyperlipidemia-related diseases.

### 4.2. Pomegranate Constituents in Diabetes Prevention

Obesity is associated with a number of chronic diseases such as type II diabetes, cardiovascular disease, chronic kidney disease, nonalcoholic fatty acid liver disease, and various types of cancers. In this regard, the effects of pomegranate in some of these disorders are also under investigation [55]. During type II diabetes, cells become increasingly insensitive to insulin. Such decreased physiological insulin levels become less effective at lowering the blood sugar levels by triggering the uptake of glucose into cells as an energy source. The levels of insulin required to elicit this same effect increase as the condition worsens, thus enhancing the insulin production in the pancreas. However, insulin resistance cannot be overcome in advanced stages of the diseases, thus resulting in extremely elevated levels of circulating glucose and insulin. Recent studies obtained promising results regarding the use of pomegranate for the treatment and prevention of type II diabetes [68, 69].

Obesity is associated with the infiltration of two phenotypically and functionally distinct subsets of macrophages (F4/80^hi^ and F4/80^lo^) into adipose tissue and a phenotypic switch from an M2 anti-inflammatory phenotype to an M1, pro-inflammatory phenotype [70]. PPAR *γ* is differentially expressed in F4/80^hi^ versus F4/80^lo^ ATM subsets, and its deficiency favors a predominance of M1 markers and impairs M2 markers expression in white adipose tissue. Moreover, the accumulation of F4/80^hi^ macrophages in adipose tissue of obese mice has also been associated with increased severity of colitis and impaired glucose tolerance [71]. Therefore, targeting macrophages arises as a therapeutic approach against obesity-related inflammation and insulin resistance. In line with these results, macrophages treated with pomegranate juice standardized to a polyphenol concentration of 75 mmol/L for 90 minutes showed a decreased degradation of oxidized LDL as well as reduced macrophage cholesterol synthesis and oxidative stress [72]. Moreover, the intake of pomegranate juice by diabetic patients results in decreased macrophage uptake of oxidized LDL and reduced serum oxidative stress, an unusually high level of oxidation that may result in damage to vital biomolecules and thereby increases disease risk [73]. Other studies showed how polyphenols and flavonoids, both of which are present in pomegranate juice, are able to inhibit LDL oxidation by the protection of LDL from cell-mediated oxidation [74, 75]. In line with these results, the modulation of PPAR*γ* by pomegranate juice resulted in reduced oxidative stress in macrophages *in vitro* [76]. However, this effect was abrogated by PPAR*γ* inhibition and was also observed after macrophages were incubated with punicalagin and gallagic acid, two of pomegranate's polyphenols. Several parts of the pomegranate have demonstrated hypoglycaemic effects *in vivo*. However, their activity profiles are slightly different. Pomegranate peel methanolic extracts containing 7.5% of gallic acid and 54.6% of ellagic acid along with other minor components did not alter insulin levels but lowered glucose levels in normoglycaemic healthy rats [77]. The administration of alloxan increased serum glucose levels and reduced serum insulin. However, alloxan administration along with pomegranate peel extract normalized such alterations [78]. Hypoglycaemic activity in streptozotocin-induced diabetes has been reported due to treatment with pomegranate seed methanol extract in rats [79]. Moreover, pomegranate seed oil has also been associated with improved insulin sensitivity in rodent animals [13, 64, 79]. Three human clinical trials have been performed with diabetic patients to assess the effect of pomegranate juice on plasma lipid and oxidation profiles. The effect derived from the administration of 50 mL of pomegranate juice per day during three months was evaluated in diabetic and healthy individuals [73]. Treated diabetic patients showed decreased serum HDL cholesterol, increased triglyceride levels, and increased values of hemoglobin A1C. Moreover, insulin levels were slightly lower in diabetic patients, and C-peptide, which is a proinsulin metabolite marker for endogenously secreted insulin, was decreased in patients' serum, thus indicating improved insulin sensitivity. Oxidative stress was decreased after a 4-week consumption of 50 mL of pomegranate juice per day [80]. This effect was attributed to increased serum HDL-associated paraoxonase/arylesterase 1 (PON1) stability and activity, an enzyme that is part of the HDL complex. Pomegranate juice concentration intake during 8 weeks showed decreased total cholesterol, LDL cholesterol, and LDL/HDL ratio [69]. Researchers did not report any adverse effect during any of these long exposures to pomegranate.

### 4.3. Modulation of Gut Inflammation by Pomegranate Seed Oil Components

Inflammation is a natural process within the innate immune response for the maintenance of normal tissue function [81]. It is the response to foreign stimuli and leads to tissue healing in normal physiological processes. However, it can become chronic and excessive inflammation if such process is dysregulated. This alteration is usual, and it is the basis for a variety of severe diseases in the gut including inflammatory bowel disease and inflammation-related colorectal cancer. PPAR*γ* and *δ* are recognized as central inhibitors of intestinal inflammation in DSS colitis [82]. Activation of PPAR*γ* and *δ* by dietary punicic acid has been shown to ameliorate intestinal inflammation in two mouse models of IBD in mice [60]. Punicic acid ameliorated DSS-induced colitis and spontaneous panenteritis in IL-10−/− mice. However, punicic acid was not effective in IL-10−/− mice with established severe inflammatory lesions such as rectal prolapses and PPAR*γ*: IL-10 DK mice. Therefore, the loss of PPAR*γ* in immune and epithelial cells impairs the ability of punicic acid to ameliorate experimental colitis. Colonic expression of PPAR*δ* and its responsive gene angiopoietin-like 4 was upregulated in IL-10−/− mice that received punicic acid preventively. Interestingly, these findings are in line with increased PPAR*δ* reporter activity induced by punicic acid *in vitro* in intestinal epithelial cells and macrophages. However, the downregulation of TNF*α* in the colons of punicic-acid-fed mice and M1 macrophages treated with punicic acid is in line with the PPAR*γ*-dependent anti-inflammatory effects of this compound. At a cellular level, the loss of PPAR*γ* in macrophages completely abrogated the anti-inflammatory activity of punicic acid, whereas its deletion in intestinal epithelial cells or the whole body deletion of PPAR*δ* impaired, but did not completely abrogate, the beneficial effects of punicic acid in the gut. Wild-type M1 classically activated macrophages showed suppressed TNF*α* and MCP-1 expression after punicic acid treatment, whereas these suppressive effects were not seen in PPAR*γ* or PPAR*δ* null macrophages. Therefore, punicic acid ameliorated IBD by downregulating inflammation in mucosal immune and epithelial cells through a PPAR*γ*- and *δ*-dependent mechanism (Figure 1).

Necrotizing colitis (NEC) is a devastating disease associated with severe and excessive intestinal inflammation, and it is the major cause of morbidity and mortality in premature infants. Although its etiology is unknown, it is thought that the combination of intestinal immaturity, high immunoreactivity of the intestinal mucosa, and genetic predisposition results in the development of NEC [83]. Gut bacteria play a crucial role in the development of immune function, and they are thought to be implicated in the predisposition and causal pathway for NEC [84]. Several studies have associated the presence of certain organisms and an increased risk of NEC [85, 86], although candidate organisms differ. Bacterial diversity in NEC pathways appears to be different from healthy individuals. Specifically, they present reduced *Firmicutes* and a bloom in Proteobacteria before NEC onset, and *Enterobacteriaceae* also seems to be associated with NEC [87]. Therefore, it seems that gut microbial contributions to NEC are mediated by changes in commensal community relationships. A recent study showed that supplementation of pomegranate seed oil into milk formula reduces the incidence and severity of NEC in rats [88]. Moreover, the administration of pomegranate seed oil protects against NEC in a neonatal rat model. Pomegranate seed oil supplementation markedly reduced the incidence of NEC from 61% to 26% and the severity of ileal damage. Since the supplementation of formula with 1.5% of pomegranate seed oil significantly increased conjugated linoleic acid levels but had no effect in other fatty acids such as CLA and arachidonic acid, the authors concluded that conjugated linoleic acid was the responsible for the protective effects of pomegranate. An increased expression of proinflammatory cytokines including IL-6, IL-8, and TNF*α* was seen in the ileum of NEC rats when compared with controls. However, the supplementation of pomegranate seed oil reduced these values. Pomegranate was also able to reduce IL-23 and IL-12, both of which contribute to intestinal inflammation by inducing a T helper 17 (Th17) and Th1 response. Therefore, pomegranate seed oil seemed to protect against NEC by downregulating the inflammatory response in the developing intestinal mucosa. Pomegranate seed oil is a rich source of fatty acids, which can be metabolized by gut microflora. Pomegranate administration activated the metabolism of punicic acid in the intestine of immature rats, thus resulting in the preservation of the intestinal architecture. Overall, pomegranate seed oil beneficial effects in NEC models are associated with improved enterocyte proliferation, protection of intestinal architecture, and reduced expression of pro-inflammatory cytokines. 

### 4.4. Anticarcinogenic Effects of Pomegranate

Colorectal cancer (CRC) is the third most commonly diagnosed cancer in the United States [89]. IBD is among the top three high-risk conditions for CRC. Among ulcerative colitis (UC) patients, one of the two main manifestations of IBD, the relative risk of developing CRC correlates with the extent and duration of disease [90]. Moreover, in patients with IBD, this risk increases by 0.5–1.0% yearly after 8–10 years [91]. In this regard, the anticarcinogenic activities of pomegranate are also under investigation. *In vitro* studies using different cancer cell lines demonstrated that pomegranate juice, seeds, and oil can inhibit cancer cell invasiveness and proliferation, cause cell cycle disruption, induce apoptosis, and inhibit tumor growth [92]. Moreover, the combination of different parts of the fruit seems to be more effective than each single extract [21]. Two studies demonstrated that pomegranate fruit extracts can inhibit cell growth and induce apoptosis by modulating the proteins that regulate apoptosis in mice implanted with prostate cancer PC-3 cell line [93, 94]. Several studies have shown a correlation between enhanced cyclooxygenase 2 (COX-2) expression and increase in cell proliferation [95]. COX-2 is a key enzyme for the conversion of arachidonic to prostaglandins, which are important inflammatory mediators. The treatment of HT-29 colon cancer cells with NS398, a COX-2 inhibitor, resulted in inhibition of proliferation [96, 97]. Pomegranate juice and punicalagin decreased COX-2 expression in HT-29 cells in a dose-dependent manner. Pomegranate juice seemed to be more effective, most likely due to significant interactions with other bioactive polyphenols such as anthocyanins and flavonols. Some studies have concluded that COX-2 expression in HT-29 cells is NF-*κ*B dependent. In this line, pomegranate juice has been shown to reduce COX-2 expression through the inhibition of phosphatidylinositide 3-kinases (PI3K) and protein kinase B or Akt, both necessary for NF-*κ*B activation [95, 98]. However, in this case, the inhibition of NF-*κ*B by wortmannin only partially decreased COX-2 expression. Therefore, other signaling pathways might be important in the modulation of COX-2 expression in HT-29 cells along with the NF-*κ*B pathways. The authors suggested the MAPK pathways (ERK1/2, p38, and JNK1,2,3) as potential candidates for this role since MAPK has been shown to mediate COX-2 expression in several studies [99, 100] (Figure 2). Pomegranate has been also shown to reduce lipoxygenase, an enzyme that catalyzes the conversion of arachidonic acid to leukotrienes, which are also key inflammatory mediators [101]. Therefore, pomegranate ingestion alters eicosanoid biosynthesis. Specifically, flavonoids extracted from pomegranate seed oil (0.015% polyphenols, 65.3% punicic acid, 4.8% palmitic acid, 2.3% stearic acid, 6.3% oleic acid, and 6.6% linoleic acid) showed a 31–44% inhibition of sheep cyclooxygenase and 69–81% inhibition of soybean lipoxygenase. Flavonoids extracted from pomegranate juice showed 21–30% inhibition of soybean lipoxygenase though no significant inhibition of sheep cyclooxygenase. Even though pomegranate juice may not be an inhibitor of cyclooxygenase-catalyzed prostaglandin formation, it may still have an indirect role in the inhibition of inflammation. Pomegranate can also suppress inflammatory cytokine expression [1, 96] as well inhibit matrix metalloproteinases (MMPs) in colon cancer cells [96, 102]. MMPs are a subgroup of collagenase enzymes expressed in arthritic joints and involved in the degradation and catabolism of extracellular joint matrix. The downregulation of MMP expression has also been seen in osteoarthritis chondrocytes pretreated with pomegranate fruit extract, thus suggesting the prevention of collagen degradation which results in joint destruction inhibition in osteoarthritis patients [103]. 

Another pro-inflammatory pathway that can be downregulated by pomegranate administration is NF-*κ*B. Constitutive activation of NF-*κ*B has been identified in some cancer cell lines [104], thus leading to inflammation and cell proliferation by the upregulating the transcription of collagenase, cell adhesion molecules, and pro-inflammatory cytokines such as TNF-*α*, IL-1, IL-2, IL-6, and IL-8 [105]. Treatment with pomegranate extracts may be successful in the therapeutic treatment of inflammatory disease and cancer since they have been shown to decrease the production of IL-6 and IL-8 and inhibit NF-*κ*B in human mast cells and basophils. The powder extract used in this study contained on average 86.0% ellagitannins, 2.5% ash, 3.2% sugars, 1.9% organic acids as citric acid equivalents, 0.8% nitrogen, and 1.2% moisture. The approximate percent distribution of pomegranate polyphenols in the extract was as follows: 19% ellagitannins as punicalagins and punicalins, 4% free ellagic acid, and 77% oligomers composed of 2–10 repeating units of gallic acid, ellagic acid, and glucose in different combinations. [106]. Moreover, treatment with urolithin A decreases certain pro-inflammatory markers including iNOS, COX-2, prostaglandin E synthase, and prostaglandin E2 in a colon cancer model [107]. Recent studies also indicate that pomegranate extracts inhibit angiogenesis by downregulating vascular endothelial growth factor in MCF-7 breast cancer and human umbilical vein endothelial cell lines. The pomegranate extracts employed in such study include pomegranate fermented juice (pomegranate “wine”) polyphenols, pomegranate pericarp polyphenols, cold-pressed pomegranate seed oil, supercritical fluid extracted pomegranate seed oil, pomegranate seed oil polyphenols, and unsaponified pomegranate seed oil fraction [108]. Another study showed how the pretreatment with dietary pomegranate oil inhibited the incidence and multiplicity of colonic adenocarcinomas in azoxymethane-induced colorectal cancer in rats [109]. Interestingly, the inhibition of tumor incidence correlated with increased expression of PPAR*γ* protein in the nontumor mucosa. All these effects described in the different studies contribute to the anti-inflammatory activity of pomegranate as well as its cancer treatment potential. Many studies have observed that the extract or the juice of pomegranate is more beneficial in terms of anticarcinogenic activity than individual or purified ingredients. Therefore, the chemical synergy when using extracts could explain the inhibition of multiple targets at the same time and consequently greater likelihood for producing cancer chemopreventive effects.

## 5. Pomegranate Safety and Drug Interactions

Pomegranate and its constituents have been safely consumed by humans for several millennia. Nevertheless, several animal studies and human clinical trials have investigated the toxicity of pomegranate. No adverse side effects have been noted in any of these studies, therefore considering safe to consume the fresh fruit or pomegranate juice in general. No toxic effects were seen after the repeated consumption of the polyphenol antioxidant punicalagin by rats, which were confirmed by histopathological analyses of their organs [31]. Pomegranate juice administration in an acute or subchronic approach resulted in no adverse effects in rats [110], and no mutagenicity or acute toxicity was seen in rats fed with pomegranate seed oil during 28 days (0 to 150,000 ppm which resulted in a mean intake of 0 to 14,214 mg/kg body weight/day) [111]. The no observable adverse effect level (NOAEL) was 4.3 g pomegranate seed oil/kg body weight/day (= 50,000 ppm). In rats and mice, the oral LD_50_ (lethal dose or dose required to kill 50% of the tested population) of pomegranate fruit extract standardized to 30% punicalagin was greater than 5 g/kg body weight, whereas the intraperitoneal LD_50_ for rats was 217 mg/kg body weight and 187 mg/kg for mice [110]. These values suggest that a healthy human individual may consume pomegranate juice, oil, or powdered extracts in moderation without high risk. In line with these results, no adverse effects or changes in renal or liver function parameters were reported during a clinical trial in 86 human overweight volunteers, which demonstrated the safety of tableted pomegranate fruit extract administration in amounts up to 1,420 mg/day during 28 days [112]. Moreover, the consumption of pomegranate juice for up to 3 years did not show any toxic effect in 10 patients with carotid artery stenosis [74]. Although any of these studies has reported any detrimental side effect, it is still not clear whether this safety extends to all extracts or pure compounds that may be used as dietary supplements in a more concentrated form.

Some studies have reported detrimental interactions between pomegranate and other drugs. For instance, recent studies have investigated the effect of pomegranate on cytochrome P450, the hepatic enzyme system involved in the metabolism of drugs and other xenobiotics along with endogenous substrates. Food-drug or drug-drug interactions can lead to the inhibition of specific CYP enzymes, thus resulting altered oral bioavailability and effectiveness or toxicity of the drug. Some studies showed that pomegranate juice inhibits cytochrome P450 enzymes CYP2C9 [113] and CYP3A [114] *in vitro* and increases levels of absorbed tolbutamide and carbamazepine, thus altering drug pharmacokinetics. A decrease in liver CYP450 and increased sleepy effects of pentobarbital was reported after a 4-week administration of pomegranate juice in mice [115]. Moreover, pomegranate juice also inhibited CYP3A using human microsomes [113, 116]. 

Although results show that a moderate intake of pomegranate is safe and does not result in any side effect, there is still a need to further study the interactions between pomegranate and other drugs as well as the effects of a long exposure and the safety of pure compounds.

## 6. Computer-Aided Drug Discovery

**Figure 3:**
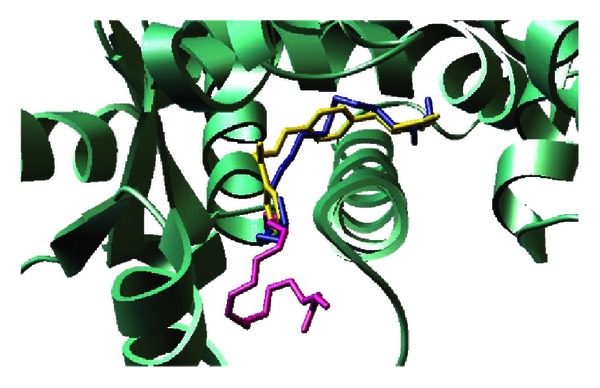
Representative binding mode of the most stable docked orientation of catalpic acid and punicic acid with peroxisome proliferator-activated receptor *γ* (PPAR*γ*). The PPAR*γ* model is shown in ribbon mode. Catalpic acid and punicic acid poses generated by AutoDock Vina are colored in blue and magenta, respectively. Rosiglitazone is colored in yellow.

Computer-aided drug discovery has become a crucial component of many drug discovery studies. Compared with the traditional physical large-scale, high-throughput screening of thematic compound libraries, which is very costly and yields mixed results, computer-aided drug discovery is more cost-effective. Structure-based virtual screening techniques have been widely used in several discovery efforts since they allow the screening of thousands of compounds within a collective of large compound libraries in a cost-effective manner [117], such as the discovery of anti-inflammatory drugs against inflammatory bowel disease [118]. The basic procedure of SBVS is to sample binding geometry for compounds from large libraries into the structure of receptor targets by using molecular modeling approaches. Each compound is sampled in thousands to millions of possible poses and scored on the basis of its complementarity to the receptor. Of the hundreds of thousands of molecules in the library, tens of top-scoring predicted ligands are subsequently tested for activity in experimental assays [119]. In an effort to expedite the mechanisms underlying the beneficial effects of pomegranate constituents, structure-based virtual screening can complement traditional experimental methods for identification of potential targets for each of these compounds. We have successfully established a protocol for screening fatty acid compounds against PPAR *γ* as a means to identify novel agonists, which can be later verified through *in vitro* assays (Figure 3) [59, 120]. Computer-aided drug discovery approaches have been used in pharmaceutical investigation for about two decades. Using reverse docking approach, researchers should be able to identify potential targets for each pomegranate compound. Moreover, structure-based virtual screening on known drug targets would provide an opportunity for *de novo* identification of natural active compounds.

## 7. Conclusions

There is strong evidence that pomegranate elicits ameliorating health effects in several diseases. When considering the pomegranate therapeutic studies along with the research investigating the bioavailability of its compounds, one can conclude that pomegranate's bioactive constituents can be absorbed and exert their biological activity. However, this fruit contains hundreds of different bioactive compounds, thus requiring a better understanding of the beneficial effects elicited by each compound and not the fruit as a whole. Moreover, some studies report that the administration of combinations of bioactive compounds has increased activity when compared to single compounds. Therefore, there is a need to further study possible synergistic effects between pomegranate's bioactive components through isobolograms in the context of ligand-binding assays and factorial designs in animal models. In this regard, the integration of computational and experimental nutritional immunology research represents a cost, and time-efficient approach for the discovery of novel interactions and mechanisms underlying such activities. 

Many of the pomegranate's beneficial effects have been widely related to the presence of ellagic acid and ellagitannins, especially punicalagins, punicalins, and gallagic acid. However, anthocyanins as well as pomegranate's distinct and unique fatty acid profile also contribute to the reported health effects. Interestingly, several effects of pomegranate are mediated by the activation of PPAR pathways by conjugated trienes derived from seed oil. Some studies suggest that pomegranate metabolites may also contribute to its therapeutic effects along with the components of the fruit. In line with these findings, pomegranate has been suggested to stimulate probiotic bacteria thus enhancing their beneficial effects and fighting bacterial infections. Therefore, gut microflora seems to be important for pomegranate therapeutic activities. Pomegranate is safe at high doses in humans. So far, pomegranate has been shown to elicit beneficial effects for the treatment of obesity, diabetes, inflammation-related diseases such as IBD and NEC, and several types of cancer, as well as cardiovascular complications. However, there is still a need to identify individual active ingredients of pomegranate as well as further explore synergistic preventive effects in laboratory, animal models, and human clinical studies. 

## Figures and Tables

**Table 1:** Chemical constituents of the pomegranate fruit: fatty acids.

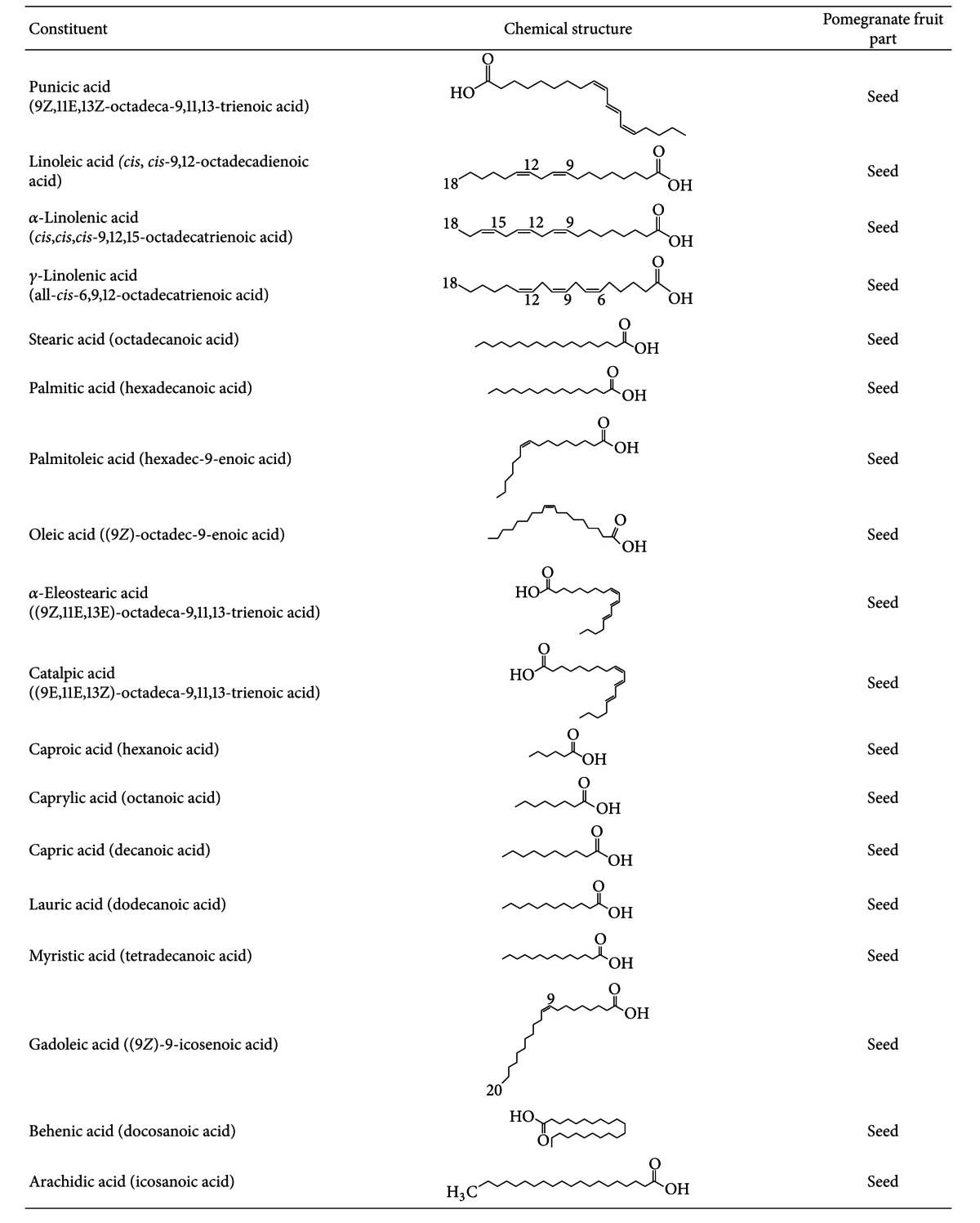

**Table 2:** Chemical constituents of the pomegranate fruit: minerals.

Constituent	Chemical structure	Pomegranate fruit part
Iron	_ 26_Fe	Seed, juice
Copper	_ 29_Cu	Seed, juice
Sodium	_ 11_Na	Seed, juice
Magnesium	_ 12_Mg	Seed, juice
Potassium	_ 19_K	Seed, juice
Calcium	_ 20_Ca	Seed, juice
Zinc	_ 30_Zn	Seed, juice
Manganese	_ 25_Mn	Seed, juice
Phosphorus	_ 15_P	Seed, juice

**Table 3:** Chemical constituents of the pomegranate fruit: Anthocyanins, tannins and phenols.

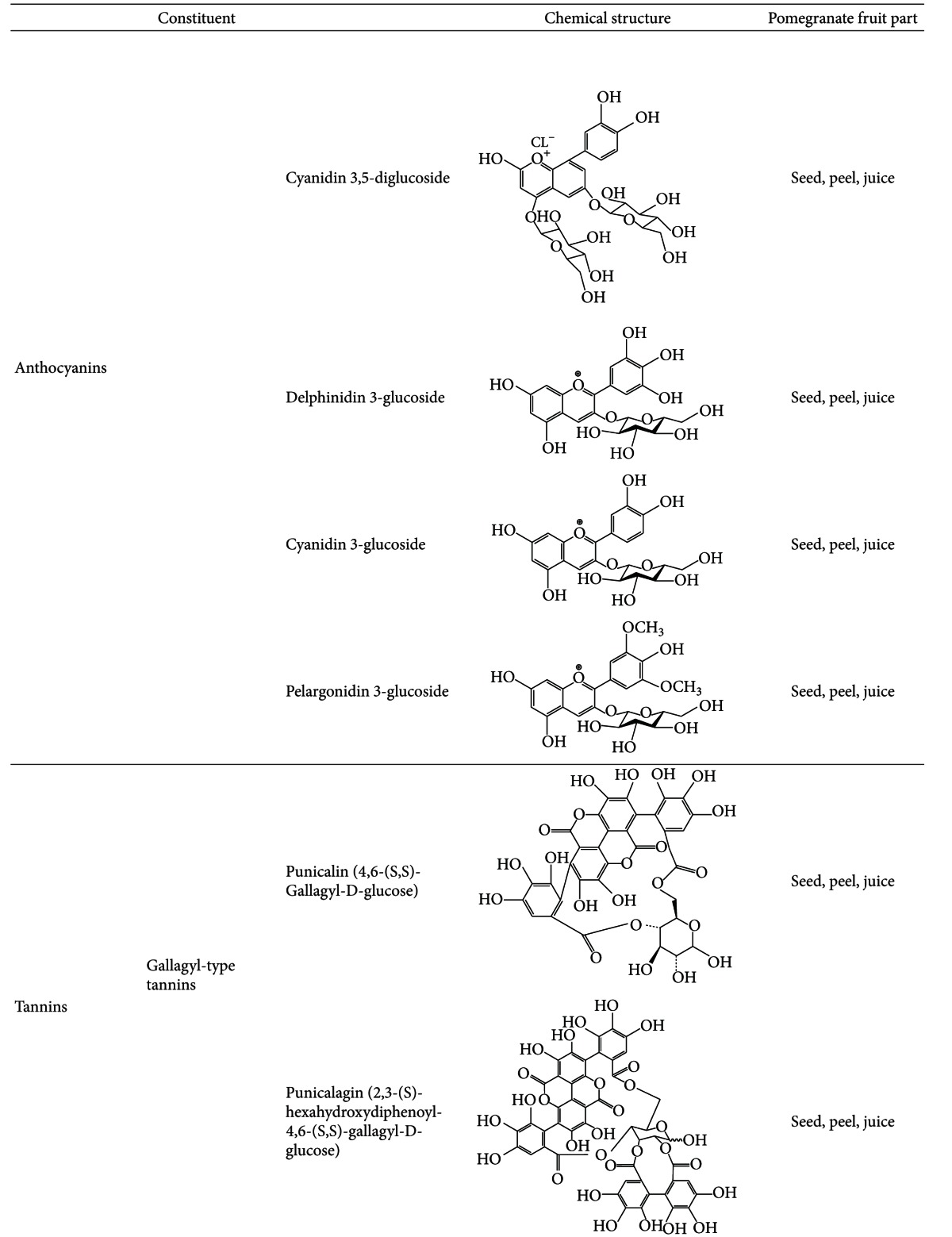 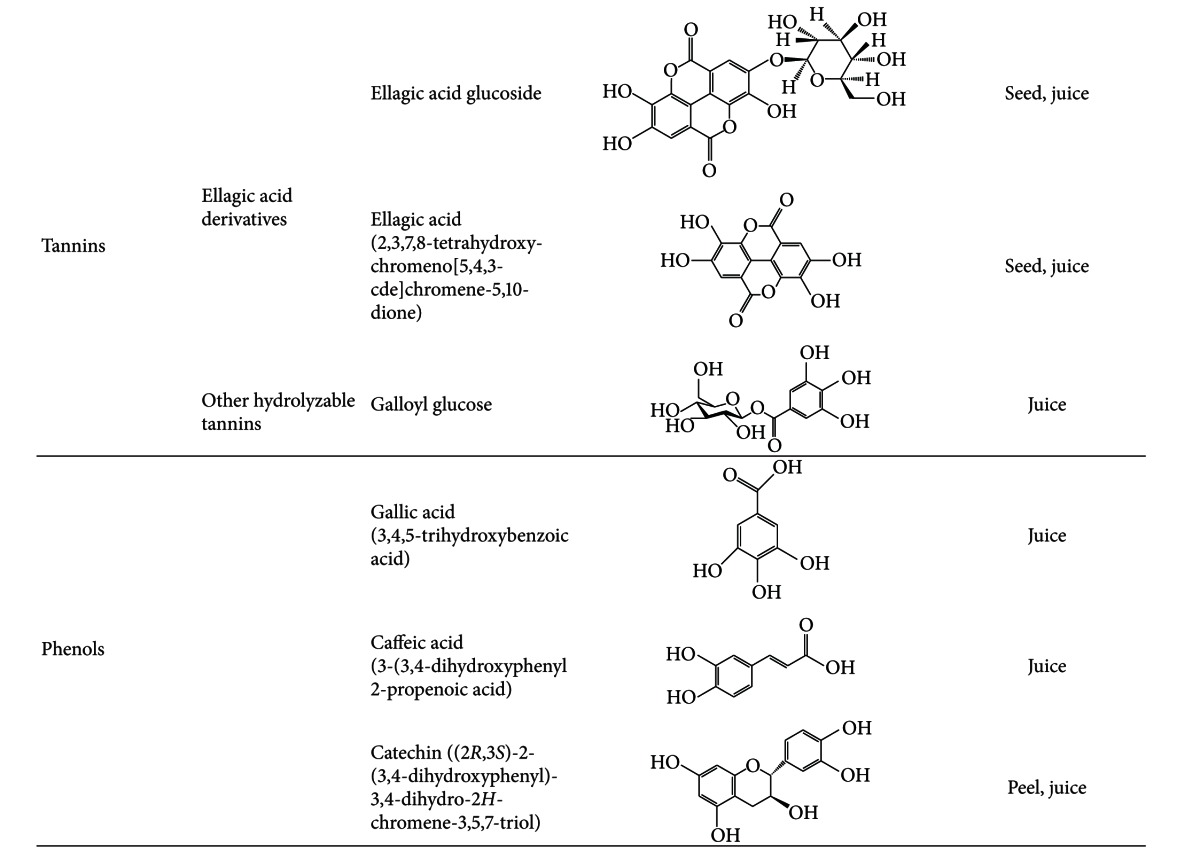

**Table 4:** Chemical constituents of the pomegranate fruit: sugars, organic acids, and antioxidants.

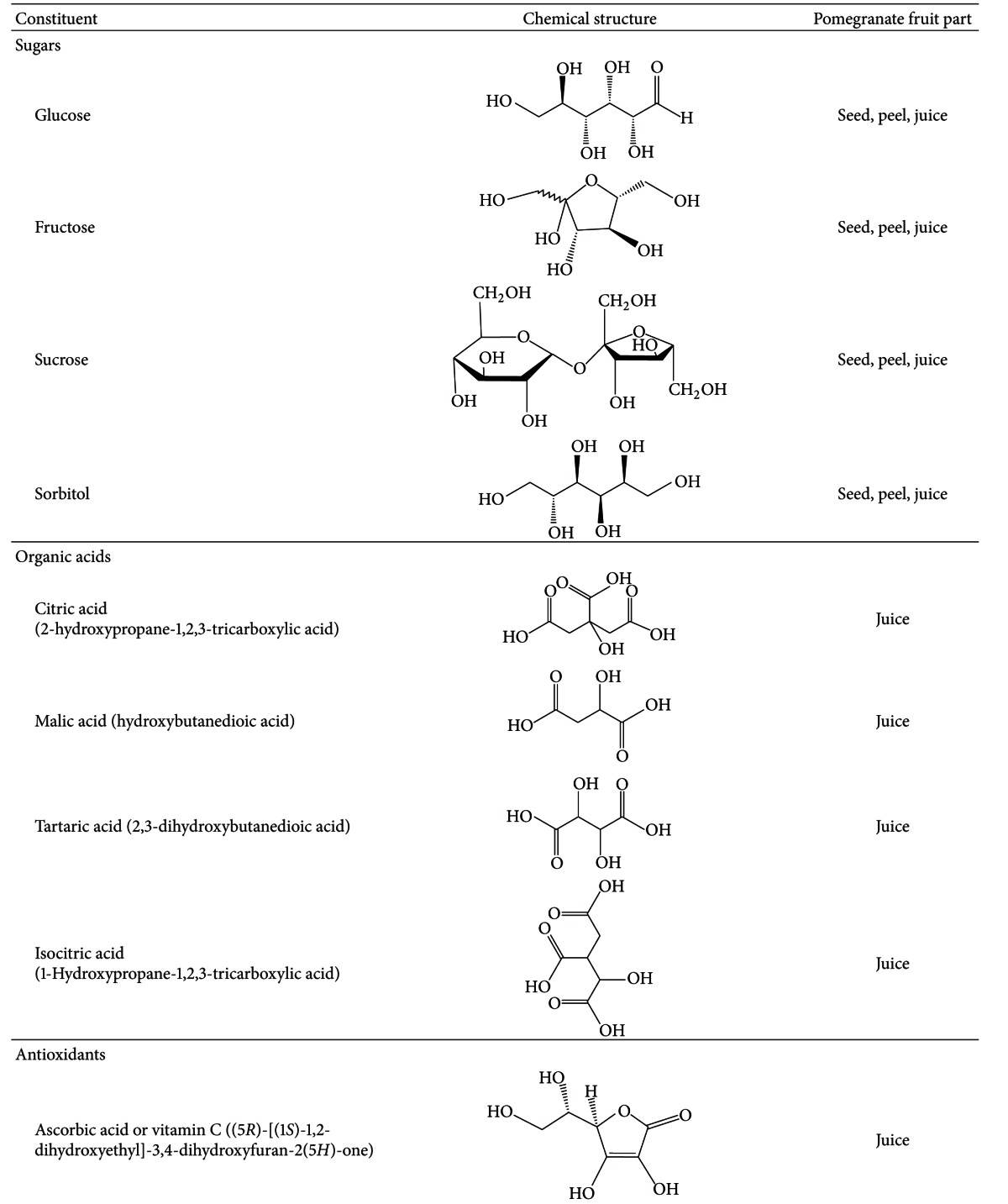 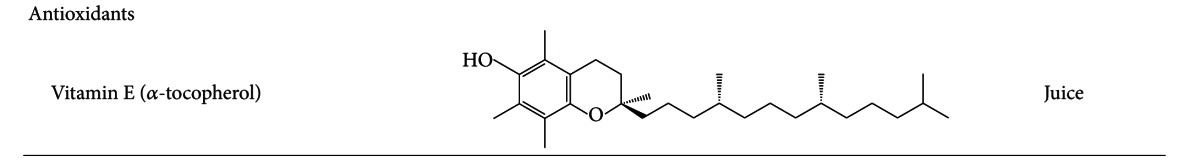

## References

[1] Lansky EP, Newman RA (2007). *Punica granatum* (pomegranate) and its potential for prevention and treatment of inflammation and cancer. *Journal of Ethnopharmacology*.

[2] Viuda-Martos M, Fernández-Lóaez J, Pérez-álvarez JA (2010). Pomegranate and its many functional components as related to human health: a review. *Comprehensive Reviews in Food Science and Food Safety*.

[3] Naqvi SAH, Khan MSY, Vohora SB (1991). Anti-bacterial, anti-fungal and anthelmintic investigations on Indian medicinal plants. *Fitoterapia*.

[4] Caceres A, Giron LM, Alvarado SR, Torres MF (1987). Screening of antimicrobial activity of plants popularly used in Guatemala for the treatment of dermatomucosal diseases. *Journal of Ethnopharmacology*.

[5] de Nigris F, Balestrieri ML, Williams-Ignarro S (2007). The influence of pomegranate fruit extract in comparison to regular pomegranate juice and seed oil on nitric oxide and arterial function in obese Zucker rats. *Nitric Oxide*.

[6] Johanningsmeier SD, Harris GK (2011). Pomegranate as a functional food and nutraceutical source. *Annual Review of Food Science and Technology*.

[7] Jurenka J (2008). Therapeutic applications of pomegranate (*Punica granatum* L.): a review. *Alternative Medicine Review*.

[8] Gil MI, Tomas-Barberan FA, Hess-Pierce B, Holcroft DM, Kader AA (2000). Antioxidant activity of pomegranate juice and its relationship with phenolic composition and processing. *Journal of Agricultural and Food Chemistry*.

[9] Li Y, Guo C, Yang J, Wei J, Xu J, Cheng S (2006). Evaluation of antioxidant properties of pomegranate peel extract in comparison with pomegranate pulp extract. *Food Chemistry*.

[10] Guo C, Yang J, Wei J, Li Y, Xu J, Jiang Y (2003). Antioxidant activities of peel, pulp and seed fractions of common fruits as determined by FRAP assay. *Nutrition Research*.

[11] Okonogi S, Duangrat C, Anuchpreeda S, Tachakittirungrod S, Chowwanapoonpohn S (2007). Comparison of antioxidant capacities and cytotoxicities of certain fruit peels. *Food Chemistry*.

[12] Sacheck J (2008). Pediatric obesity: an inflammatory condition?. *Journal of Parenteral and Enteral Nutrition*.

[13] Vroegrijk IOCM, van Diepen JA, van den Berg S (2011). Pomegranate seed oil, a rich source of punicic acid, prevents diet-induced obesity and insulin resistance in mice. *Food and Chemical Toxicology*.

[14] He J, Giusti MM (2010). Anthocyanins: natural colorants with health-promoting properties. *Annual Review of Food Science and Technology*.

[15] Basu A, Penugonda K (2009). Pomegranate juice: a heart-healthy fruit juice. *Nutrition Reviews*.

[16] Stowe CB (2011). The effects of pomegranate juice consumption on blood pressure and cardiovascular health. *Complementary Therapies in Clinical Practice*.

[17] Singh RP, Murthy KNC, Jayaprakasha GK (2002). Studies on the antioxidant activity of pomegranate (*Punica granatum*) peel and seed extracts using *in vitro* models. *Journal of Agricultural and Food Chemistry*.

[18] Kulkarni AP, Mahal HS, Kapoor S, Aradhya SM (2007). *In vitro* studies on the binding, antioxidant, and cytotoxic action of punicalagin. *Journal of Agricultural and Food Chemistry*.

[19] Falsaperla M, Morgia G, Tartarone A, Ardito R, Romano G (2005). Support ellagic acid therapy in patients with hormone refractory prostate cancer (HRPC) on standard chemotherapy using vinorelbine and estramustine phosphate. *European Urology*.

[20] Hassoun EA, Vodhanel J, Abushaban A (2004). The modulatory effects of ellagic acid and vitamin E succinate on TCDD-induced oxidative stress in different brain regions of rats after subchronic exposure. *Journal of Biochemical and Molecular Toxicology*.

[21] Lansky EP, Jiang W, Mo H (2005). Possible synergistic prostate cancer suppression by anatomically discrete pomegranate fractions. *Investigational New Drugs*.

[22] Lansky EP, Harrison G, Froom P, Jiang WG (2005). Pomegranate (*Punica granatum*) pure chemicals show possible synergistic inhibition of human PC-3 prostate cancer cell invasion across Matrigel. *Investigational New Drugs*.

[23] Mirdehghan SH, Rahemi M (2007). Seasonal changes of mineral nutrients and phenolics in pomegranate (*Punica granatum* L.) fruit. *Scientia Horticulturae*.

[24] Seeram NP, Lee R, Heber D (2004). Bioavailability of ellagic acid in human plasma after consumption of ellagitannins from pomegranate (*Punica granatum* L.) juice. *Clinica Chimica Acta*.

[25] Seeram NP, Henning SM, Zhang Y, Suchard M, Li Z, Heber D (2006). Pomegranate juice ellagitannin metabolites are present in human plasma and some persist in urine for up to 48 hours. *Journal of Nutrition*.

[26] González-Sarrías A, Larrosa M, Toms-Barberán FA, Dolara P, Espín JC (2010). NF-*κ*B-dependent anti-inflammatory activity of urolithins, gut microbiota ellagic acid-derived metabolites, in human colonic fibroblasts. *British Journal of Nutrition*.

[27] Dobroslawa B, Kasimsetty SG, Khan SI, Daneel F (2009). Urolithins, intestinal microbial metabolites of pomegranate ellagitannins, exhibit potent antioxidant activity in a cell-based assay. *Journal of Agricultural and Food Chemistry*.

[28] Seeram NP, Zhang Y, McKeever R (2008). Pomegranate juice and extracts provide similar levels of plasma and urinary ellagitannin metabolites in human subjects. *Journal of Medicinal Food*.

[29] Cerdá B, Espín JC, Parra S, Martínez P, Tomás-Barberán FA (2004). The potent *in vitro* antioxidant ellagitannins from pomegranate juice are metabolised into bioavailable but poor antioxidant hydroxy-6H-dibenzopyran-6-one derivatives by the colonic microflora of healthy humans. *European Journal of Nutrition*.

[30] Cerdá B, Llorach R, Cerón JJ, Espín JC, Tomás-Barberán FA (2003). Evaluation of the bioavailability and metabolism in the rat of punicalagin, an antioxidant polyphenol from pomegranate juice. *European Journal of Nutrition*.

[31] Cerdá B, Cerón JJ, Tomás-Barberán FA, Espín JC (2003). Repeated oral administration of high doses of the pomegranate ellagitannin punicalagin to rats for 37 days is not toxic. *Journal of Agricultural and Food Chemistry*.

[32] Mertens-Talcott SU, Jilma-Stohlawetz P, Rios J, Hingorani L, Derendorf H (2006). Absorption, metabolism, and antioxidant effects of pomegranate (*Punica granatum* L.) polyphenols after ingestion of a standardized extract in healthy human volunteers. *Journal of Agricultural and Food Chemistry*.

[33] Espín JC, González-Barrio R, Cerdá B, López-Bote C, Rey AI, Tomás-Barberán FA (2007). Iberian pig as a model to clarify obscure points in the bioavailability and metabolism of ellagitannins in humans. *Journal of Agricultural and Food Chemistry*.

[34] Seeram NP, Aronson WJ, Zhang Y (2007). Pomegranate ellagitannin-derived metabolites inhibit prostate cancer growth and localize to the mouse prostate gland. *Journal of Agricultural and Food Chemistry*.

[35] González-Sarrías A, Giménez-Bastida JA, García-Conesa MT (2010). Occurrence of urolithins, gut microbiota ellagic acid metabolites and proliferation markers expression response in the human prostate gland upon consumption of walnuts and pomegranate juice. *Molecular Nutrition and Food Research*.

[36] Crawford PA, Gordon JI (2005). Microbial regulation of intestinal radiosensitivity. *Proceedings of the National Academy of Sciences of the United States of America*.

[37] Bialonska D, Ramnani P, Kasimsetty SG, Muntha KR, Gibson GR, Ferreira D (2010). The influence of pomegranate by-product and punicalagins on selected groups of human intestinal microbiota. *International Journal of Food Microbiology*.

[38] Gibson GR (2008). Prebiotics as gut microflora management tools. *Journal of Clinical Gastroenterology*.

[39] Rastall RA, Gibson GR, Gill HS (2005). Modulation of the microbial ecology of the human colon by probiotics, prebiotics and synbiotics to enhance human health: an overview of enabling science and potential applications. *FEMS Microbiology Ecology*.

[40] Bialonska D, Kasimsetty SG, Schrader KK, Ferreira D (2009). The effect of pomegranate (*Punica granatum* L.) byproducts and ellagitannins on the growth of human gut bacteria. *Journal of Agricultural and Food Chemistry*.

[41] Mokdad AH, Bowman BA, Ford ES, Vinicor F, Marks JS, Koplan JP (2001). The continuing epidemics of obesity and diabetes in the United States. *Journal of the American Medical Association*.

[42] Desvergne B, Wahli W (1999). Peroxisome proliferator-activated receptors: nuclear control of metabolism. *Endocrine Reviews*.

[43] Nesto RW, Bell D, Bonow RO (2004). Thiazolidinedione use, fluid retention, and congestive heart failure: a consensus statement from the American Heart Association and American Diabetes Association. *Diabetes Care*.

[44] Katayama K, Wada K, Nakajima A (2003). A novel PPAR*γ* gene therapy to control inflammation associated with inflammatory bowel disease in a murine model. *Gastroenterology*.

[45] Dubuquoy L, Å Jansson E, Deeb S (2003). Impaired expression of peroxisome proliferator-activated receptor *γ*in ulcerative colitis. *Gastroenterology*.

[46] Ogata T, Miyauchi T, Sakai S, Irukayama-Tomobe Y, Goto K, Yamaguchi I (2002). Stimulation of peroxisome-proliferator-activated receptor *α* (PPAR*α*) attenuates cardiac fibrosis and endothelin-1 production in pressure-overloaded rat hearts. *Clinical Science*.

[47] Hsu SC, Huang CJ (2007). Changes in liver PPAR*α* mRNA expression in response to two levels of high-safflower-oil diets correlate with changes in adiposity and serum leptin in rats and mice. *Journal of Nutritional Biochemistry*.

[48] Daynes RA, Jones DC (2002). Emerging roles of PPARs in inflammation and immunity. *Nature Reviews Immunology*.

[49] Mangelsdorf DJ, Thummel C, Beato M (1995). The nuclear receptor super-family: the second decade. *Cell*.

[50] Chinetti G, Lestavel S, Bocher V (2001). PPAR-*α* and PPAR-*γ* activators induce cholesterol removal from human macrophage foam cells through stimulation of the ABCA1 pathway. *Nature Medicine*.

[51] Park BH, Vogelstein B, Kinzler KW (2001). Genetic disruption of PPAR*δ* decreases the tumorigenicity of human colon cancer cells. *Proceedings of the National Academy of Sciences of the United States of America*.

[52] Ricote M, Li AC, Willson TM, Kelly CJ, Glass CK (1998). The peroxisome proliferator-activated receptor-*γ* is a negative regulator of macrophage activation. *Nature*.

[53] Wahli W (2008). A gut feeling of the PXR, PPAR and NF-*κ*B connection. *Journal of Internal Medicine*.

[54] Bassaganya-Riera J, Reynolds K, Martino-Catt S (2004). Activation of PPAR *γ* and *δ* by conjugated linoleic acid mediates protection from experimental inflammatory bowel disease. *Gastroenterology*.

[55] Bassaganya-Riera J, Guri AJ, Hontecillas R (2011). Treatment of obesity-related complications with novel classes of naturally occurring PPAR agonists. *Journal of Obesity*.

[56] Lewis JD, Lichtenstein GR, Deren JJ (2008). Rosiglitazone for active ulcerative colitis: a randomized placebo-controlled trial. *Gastroenterology*.

[57] Elasy TA, Griffin M (2004). Thiazolidinedione use, fluid retention, and congestive heart failure: a consensus statement from the American Heart Association and American Diabetes Association: response to Nesto. *Diabetes Care*.

[58] Bassaganya-Riera J, Viladomiu M, Pedragosa M (2012). Probiotic bacteria produce conjugated linoleic acid locally in the gut that targets macrophage PPAR gamma to suppress colitis. *PLoS One*.

[59] Lewis SN, Brannan L, Guri AJ (2011). Dietary alpha-eleostearic acid ameliorates experimental inflammatory bowel disease in mice by activating peroxisome proliferator-activated receptor-gamma. *PLoS One*.

[60] Bassaganya-Riera J, DiGuardo M, Climent M (2011). Activation of PPARgamma and delta by dietary punicic acid ameliorates intestinal inflammation in mice. *British Journal of Nutrition*.

[61] Tenenbaum A, Motro M, Fisman EZ (2005). Dual and pan-peroxisome proliferator-activated receptors (PPAR) co-agonism: the bezafibrate lessons. *Cardiovascular Diabetology*.

[62] Hontecillas R, O’Shea M, Einerhand A, Diguardo M, Bassaganya-Riera J (2009). Activation of PPAR *γ* and *α* by punicic acid ameliorates glucose tolerance and suppresses obesity-related inflammation. *Journal of the American College of Nutrition*.

[63] Boussetta T, Raad H, Lettéron P (2009). Punicic acid a conjugated linolenic acid inhibits TNF*α*-induced neutrophil hyperactivation and protects from experimental colon inflammation in rats. *PLoS ONE*.

[64] McFarlin BK, Strohacker KA, Kueht ML (2009). Pomegranate seed oil consumption during a period of high-fat feeding reduces weight gain and reduces type 2 diabetes risk in CD-1 mice. *British Journal of Nutrition*.

[65] Hontecillas R, Diguardo M, Duran E, Orpi M, Bassaganya-Riera J (2008). Catalpic acid decreases abdominal fat deposition, improves glucose homeostasis and upregulates PPAR *α* expression in adipose tissue. *Clinical Nutrition*.

[66] Aasum E, Belke DD, Severson DL (2002). Cardiac function and metabolism in type 2 diabetic mice after treatment with BM 17.0744, a novel PPAR-*α* activator. *American Journal of Physiology*.

[67] Jang A, Srinivasan P, Lee NY (2008). Comparison of hypolipidemic activity of synthetic gallic acid-linoleic acid ester with mixture of gallic acid and linoleic acid, gallic acid, and linoleic acid on high-fat diet induced obesity in C57BL/6 Cr Slc mice. *Chemico-Biological Interactions*.

[68] Esmaillzadeh A, Tahbaz F, Gaieni I, Alavi-Majd H, Azadbakht L (2006). Cholesterol-lowering effect of concentrated pomegranate juice consumption in type II diabetic patients with hyperlipidemia. *International Journal for Vitamin and Nutrition Research*.

[69] Esmaillzadeh A, Tahbaz F, Gaieni I, Alavi-Majd H, Azadbakht L (2004). Concentrated pomegranate juice improves lipid profiles in diabetic patients with hyperlipidemia. *Journal of Medicinal Food*.

[70] Bassaganya-Riera J, Misyak S, Guri AJ, Hontecillas R (2009). PPAR*γ* is highly expressed in F4/80hi adipose tissue macrophages and dampens adipose-tissue inflammation. *Cellular Immunology*.

[71] Guri AJ, Hontecillas R, Ferrer G (2008). Loss of PPAR*γ* in immune cells impairs the ability of abscisic acid to improve insulin sensitivity by suppressing monocyte chemoattractant protein-1 expression and macrophage infiltration into white adipose tissue. *Journal of Nutritional Biochemistry*.

[72] Fuhrman B, Volkova N, Aviram M (2005). Pomegranate juice inhibits oxidized LDL uptake and cholesterol biosynthesis in macrophages. *Journal of Nutritional Biochemistry*.

[73] Rosenblat M, Hayek T, Aviram M (2006). Anti-oxidative effects of pomegranate juice (PJ) consumption by diabetic patients on serum and on macrophages. *Atherosclerosis*.

[74] Aviram M, Dornfeld L, Rosenblat M (2000). Pomegranate juice consumption reduces oxidative stress, atherogenic modifications to LDL, and platelet aggregation: studies in humans and in atherosclerotic apolipoprotein E-deficient mice. *American Journal of Clinical Nutrition*.

[75] Aviram M, Dornfeld L, Kaplan M (2002). Pomegranate juice flavonoids inhibit low-density lipoprotein oxidation and cardiovascular diseases: studies in atherosclerotic mice and in humans. *Drugs under Experimental and Clinical Research*.

[76] Shiner M, Fuhrman B, Aviram M (2007). Macrophage paraoxonase 2 (PON2) expression is up-regulated by pomegranate juice phenolic anti-oxidants via PPAR*γ* and AP-1 pathway activation. *Atherosclerosis*.

[77] Parmar HS, Kar A (2008). Medicinal values of fruit peels from Citrus sinensis, *Punica granatum*, and Musa paradisiaca with respect to alterations in tissue lipid peroxidation and serum concentration of glucose, insulin, and thyroid hormones. *Journal of Medicinal Food*.

[78] Parmar HS, Kar A (2007). Antidiabetic potential of Citrus sinensis and *Punica granatum* peel extracts in alloxan treated male mice. *BioFactors*.

[79] Abazov VM, Abbott B, Abolins M (2008). Search for neutral higgs bosons in Multi-*b*-Jet events in $p\bar{p}$ collisions at $\sqrt{s}=1.96$TeV. *Physical Review Letters*.

[80] Rock W, Rosenblat M, Miller-Lotan R, Levy AP, Elias M, Aviram M (2008). Consumption of Wonderful variety pomegranate juice and extract by diabetic patients increases paraoxonase 1 association with high-density lipoprotein and stimulates its catalytic activities. *Journal of Agricultural and Food Chemistry*.

[81] Medjakovic S, Jungbauer A (2013). Pomegranate: a fruit that ameliorates metabolic syndrome. *Food & Function*.

[82] Nakajima A, Wada K, Katayama K (2002). Gene expression profile after peroxisome proliferator activator receptor-*γ* ligand administration in dextran sodium sulfate mice. *Journal of Gastroenterology*.

[83] Carlisle EM, Poroyko V, Caplan MS, Alverdy JA, Liu D (2011). Gram negative bacteria are associated with the early stages of necrotizing enterocolitis. *PLoS ONE*.

[84] Morowitz MJ, Poroyko V, Caplan M, Alverdy J, Liu DC (2010). Redefining the role of intestinal microbes in the pathogenesis of necrotizing enterocolitis. *Pediatrics*.

[85] Meijer CMMW, Degener JE, Dzoljic Danilovic G (1983). Quantitative study of the aerobic and anaerobic faecal flora in neonatal necrotising enterocolitis. *Archives of Disease in Childhood*.

[86] Hoy C, Millar MR, MacKay P, Godwin PGR, Langdale V, Levene MI (1990). Quantitative changes in faecal microflora preceding necrotising enterocolitis in premature neonates. *Archives of Disease in Childhood*.

[87] Mai V, Young CM, Ukhanova M (2011). Fecal microbiota in premature infants prior to necrotizing enterocolitis. *PLoS ONE*.

[88] Coursodon-Boyiddle CF, Snarrenberg CL, Adkins-Rieck CK (2012). Pomegranate seed oil reduces intestinal damage in a rat model of necrotizing enterocolitis. *American Journal of Physiology*.

[89] Jemal A, Siegel R, Ward E (2008). Cancer statistics, 2008. *CA Cancer Journal for Clinicians*.

[90] Eaden JA, Abrams KR, Mayberry JF (2001). The risk of colorectal cancer in ulcerative colitis: a meta-analysis. *Gut*.

[91] Munkholm P (2003). Review article: the incidence and prevalence of colorectal cancer in inflammatory bowel disease. *Alimentary Pharmacology and Therapeutics*.

[92] Albrecht M, Jiang W, Kumi-Diaka J (2004). Pomegranate extracts potently suppress proliferation, xenograft growth, and invasion of human prostate cancer cells. *Journal of Medicinal Food*.

[93] Malik A, Mukhtar H (2006). Prostate cancer prevention through pomegranate fruit. *Cell Cycle*.

[94] Malik A, Afaq F, Sarfaraz S, Adhami VM, Syed DN, Mukhtar H (2005). Pomegranate fruit juice for chemoprevention and chemotherapy of prostate cancer. *Proceedings of the National Academy of Sciences of the United States of America*.

[95] Meei LS, Kuo FC, Yen JS, Wan WL, Shoei YLS, Shing HL (2005). Activation of phosphoinositide 3-kinase in response to inflammation and nitric oxide leads to the up-regulation of cyclooxygenase-2 expression and subsequent cell proliferation in mesangial cells. *Cellular Signalling*.

[96] Adams LS, Seeram NP, Aggarwal BB, Takada Y, Sand D, Heber D (2006). Pomegranate juice, total pomegranate ellagitannins, and punicalagin suppress inflammatory cell signaling in colon cancer cells. *Journal of Agricultural and Food Chemistry*.

[97] Shukla S, Gupta S (2004). Molecular mechanisms for apigenin-induced cell-cycle arrest and apoptosis of hormone refractory human prostate carcinoma DU145 cells. *Molecular Carcinogenesis*.

[98] Matsuura H, Sakaue M, Subbaramaiah K (1999). Regulation of cyclooxygenase-2 by interferon *γ* and transforming growth factor *α* in normal human epidermal keratinocytes and squamous carcinoma cells. Role of mitogen-activated protein kinases. *Journal of Biological Chemistry*.

[99] Subbaramaiah K, Dannenberg AJ (2003). Cyclooxygenase 2: a molecular target for cancer prevention and treatment. *Trends in Pharmacological Sciences*.

[100] Afaq F, Saleem M, Krueger CG, Reed JD, Mukhtar H (2005). Anthocyanin- and hydrolyzable tannin-rich pomegranate fruit extract modulates MAPK and NF-*κ*B pathways and inhibits skin tumorigenesis in CD-1 mice. *International Journal of Cancer*.

[101] Schubert SY, Lansky EP, Neeman I (1999). Antioxidant and eicosanoid enzyme inhibition properties of pomegranate seed oil and fermented juice flavonoids. *Journal of Ethnopharmacology*.

[102] Okamoto T, Akuta T, Tamura F, van der Vliet A, Akaike T (2004). Molecular mechanism for activation and regulation of matrix metalloproteinases during bacterial infections and respiratory inflammation. *Biological Chemistry*.

[103] Ahmed S, Wang N, Hafeez BB, Cheruvu VK, Haqqi TM (2005). *Punica granatum* L. extracts inhibits IL-1*β*-induced expression of matrix metalloproteinases by inhibiting the activation of MAP kinases and NF-*κ*B in human chondrocytes *in vitro*. *Journal of Nutrition*.

[104] Heber D (2008). Multitargeted therapy of cancer by ellagitannins. *Cancer Letters*.

[105] Conner EM, Grisham MB (1996). Inflammation, free radicals and antioxidants. *Nutrition*.

[106] Rasheed Z, Akhtar N, Anbazhagan AN, Ramamurthy S, Shukla M, Haqqi TM (2009). Polyphenol-rich pomegranate fruit extract (POMx) suppresses PMACI-induced expression of pro-inflammatory cytokines by inhibiting the activation of MAP kinases and NF-*κ*B in human KU812 cells. *Journal of Inflammation*.

[107] Larrosa M, González-Sarrías A, Yáñez-Gascón MJ (2010). Anti-inflammatory properties of a pomegranate extract and its metabolite urolithin-A in a colitis rat model and the effect of colon inflammation on phenolic metabolism. *Journal of Nutritional Biochemistry*.

[108] Toi M, Bando H, Ramachandran C (2003). Preliminary studies on the anti-angiogenic potential of pomegranate fractions *in vitro* and *in vivo*. *Angiogenesis*.

[109] Kohno H, Suzuki R, Yasui Y, Hosokawa M, Miyashita K, Tanaka T (2004). Pomegranate seed oil rich in conjugated linolenic acid suppresses chemically induced colon carcinogenesis in rats. *Cancer Science*.

[110] Patel C, Dadhaniya P, Hingorani L, Soni MG (2008). Safety assessment of pomegranate fruit extract: acute and subchronic toxicity studies. *Food and Chemical Toxicology*.

[111] Meerts IA, Verspeek-Rip CM, Buskens CA (2009). Toxicological evaluation of pomegranate seed oil. *Food and Chemical Toxicology*.

[112] Heber D, Seeram NP, Wyatt H (2007). Safety and antioxidant activity of a pomegranate ellagitannin-enriched polyphenol dietary supplement in overweight individuals with increased waist size. *Journal of Agricultural and Food Chemistry*.

[113] Nagata M, Hidaka M, Sekiya H (2007). Effects of pomegranate juice on human cytochrome P450 2C9 and tolbutamide pharmacokinetics in rats. *Drug Metabolism and Disposition*.

[114] Hidaka M, Okumura M, Fujita KI (2005). Effects of pomegranate juice on human cytochrome P450 3A (CYP3A) and carbamazepine pharmacokinetics in rats. *Drug Metabolism and Disposition*.

[115] Faria A, Monteiro R, Azevedo I, Calhau C (2007). Pomegranate juice effects on cytochrome p450s expression: *in vivo* studies. *Journal of Medicinal Food*.

[116] Kim H, Yoon YJ, Shon JH, Cha IJ, Shin JG, Liu KH (2006). Inhibitory effects of fruit juices on CYP3A activity. *Drug Metabolism and Disposition*.

[117] Klebe G (2006). Virtual ligand screening: strategies, perspectives and limitations. *Drug Discovery Today*.

[118] Lu P, Hontecillas R, Horne WT (2012). Computational modeling-based discovery of novel classes of anti-inflammatory drugs that target lanthionine synthetase C-like protein 2. *PLoS One*.

[119] Shoichet BK (2004). Virtual screening of chemical libraries. *Nature*.

[120] Lewis SN, Bassaganya-Riera J, Bevan DR (2010). Virtual screening as a technique for PPAR modulator discovery. *PPAR Research*.

